# Daycare Attendance, Breastfeeding, and the Development of Type 1 Diabetes: The Diabetes Autoimmunity Study in the Young

**DOI:** 10.1155/2015/203947

**Published:** 2015-03-25

**Authors:** Katelyn Hall, Brittni Frederiksen, Marian Rewers, Jill M. Norris

**Affiliations:** ^1^Department of Epidemiology, Colorado School of Public Health, University of Colorado Denver, Aurora, CO 80045, USA; ^2^The Barbara Davis Center for Diabetes, University of Colorado Denver, Aurora, CO 80045, USA

## Abstract

*Background.* The hygiene hypothesis attributes the increased incidence of type 1 diabetes (T1D) to a decrease of immune system stimuli from infections. We evaluated this prospectively in the Diabetes Autoimmunity Study in the Young (DAISY) by examining daycare attendance during the first two years of life (as a proxy for infections) and the risk of T1D. *Methods.* DAISY is a prospective cohort of children at increased T1D risk. Analyses were limited to 1783 children with complete daycare and breastfeeding data from birth to 2 years of age; 58 children developed T1D. Daycare was defined as supervised time with at least one other child at least 3 times a week. Breastfeeding duration was evaluated as a modifier of the effect of daycare. Cox proportional hazards regression was used for analyses. *Results.* Attending daycare before the age of 2 years was not associated with T1D risk (HR: 0.89; CI: 0.54–1.47) after adjusting for HLA, first degree relative with T1D, ethnicity, and breastfeeding duration. Breastfeeding duration modified this association, where daycare attendance was associated with increased T1D risk in nonbreastfed children and a decreasing T1D risk with increasing breastfeeding duration (interaction *P *value = 0.02). *Conclusions.* These preliminary data suggest breastfeeding may modify the effect of daycare on T1D risk.

## 1. Background

Type 1 diabetes (T1D) is an autoimmune disease where the body's immune system destroys the pancreatic beta cells that produce insulin. The incidence of T1D is increasing at roughly 3% globally, with the greatest increase of incidence in children younger than 4 years of age [1]. It is likely that an individual with the genetic makeup for diabetes will not develop T1D without an immunologic trigger that initiates the autoimmune response [2]. While the autoimmune pathophysiology of T1D has been established, a deeper understanding of this trigger has remained elusive.

The hygiene hypothesis proposes that the recent increase in incidence of T1D is due to increased hygiene and low pathogen burden environments [3]. Exposures to infectious agents early in life are hypothesized to activate regulatory pathways in our immune system that suppress development of autoimmunity and thus T1D [4]. Social mixing is a variable used to encompass the numerous exposures to infectious agents that individuals experience when sharing space together. Social mixing captures asymptomatic or minor infections that would otherwise not be reported or recalled. Previous studies used social mixing as a proxy for infections to test the hygiene hypothesis and have observed lower risk of T1D in high social mixing environments [5, 6]. Parslow et al. observed a significant association with higher incidence of T1D for children 0–14 years of age in areas with low levels of social mixing [7]. In Scotland, Patterson and Waugh examined social mixing socioeconomically and geographically and found that incidence of T1D was lower in deprived urban areas compared with affluent rural areas [8]. In Austria, Schober et al. examined social mixing through population density and observed protection from T1D in areas with high percentages of children less than 15 years of age [5].

Daycare offers social mixing during critical immune development stages early in life. Like social mixing, attending daycare can be used as a proxy for measuring asymptomatic or minor infections to test the hygiene hypothesis. McKinney et al. found evidence that social mixing through daycare attendance early in life protected against the development of T1D [6]. A meta-analysis of several case-control studies showed a statistically significant protective effect of daycare on the risk of T1D [9]. The previous studies examining daycare attendance and the risk of developing T1D have been retrospective; and the authors have recommended that future studies analyze this association prospectively. This study will attempt to close the gap on the lack of prospective analysis by examining daycare attendance and the risk of developing T1D prospectively using the Diabetes Autoimmunity Study in the Young (DAISY) cohort.

Breastfeeding has also been shown to be protective in the risk of developing T1D, albeit inconsistently [10, 11]. It is believed that breastfeeding provides immune support through immunoglobulin A antibodies and increased *β*-cell proliferation [12] to protect against infections and thus reduce the risk of T1D.

We hypothesized that daycare attendance is associated with a decreased risk of developing T1D in children in DAISY. We further hypothesized that the effect of daycare attendance is modified by breastfeeding.

## 2. Methods

### 2.1. Study Population

DAISY is a prospective study of children in Colorado who are at increased risk of developing T1D. It includes children born at St. Joseph's Hospital in Denver that were screened by umbilical cord blood for diabetes-susceptibility alleles in the human leukocyte antigen (HLA) region. It also includes unaffected children recruited between birth and 8 years of age with a first degree relative that has T1D. For these analyses, we included only the DAISY children who had a clinic visit before 1.35 years of age and who had prospective daycare exposure data from birth until two years of age and complete breastfeeding duration data. Interviews collecting diet and daycare data were completed at 3, 6, 9, 12, 15, and 24 months and then annually thereafter. Clinic visits occurred at 9, 15, and 24 months and annually thereafter for the tracking of autoimmunity and T1D.

The following descriptive factors were examined: HLA genotype (HLA-DR3/4, DQB1^*^0302 versus others), first degree relative with T1D (mother versus father or sibling versus none), birth order (first/only child versus second child or more), sex (female versus male), race/ethnicity (non-Hispanic white versus other race/ethnicity), maternal age at child's birth, maternal education (>12 years versus ≤12 years), crowding (≥1 person/room versus <1 person/room at 6 months of age), and breastfeeding duration (in months). Crowding was calculated by taking the reported number of persons living in a household and dividing this by the number of rooms in the household, not including bathrooms, when the child was six months of age.

### 2.2. Daycare Measure

Daycare information was collected by parent interview with the following query, “Does ___ attend daycare (family daycare home or daycare center) or preschool on a regular basis?” Follow-up questions regarding the size of the daycare/preschool class and the frequency of attendance were asked. The daycare variable used in this study was defined as supervised time with at least one other child, not including a sibling, at least three times a week.

### 2.3. Breastfeeding Duration Measure

Breastfeeding duration was defined as the length of time, in months, that the child was breastfed, either partially or exclusively.

### 2.4. Diagnosis of Type 1 Diabetes

T1D was diagnosed by a physician based on symptoms of excessive urination and/or excessive thirst with at least a glucose level greater than 200 mg/dL, a fasting plasma glucose level at or above 126 mg/dL, or an oral glucose tolerance test with a 2-hour glucose level at or above 200 mg/dL.

### 2.5. Analysis Population

Of the 2,632 children followed by DAISY, 1,856 children were followed from birth; that is, they had a clinic visit before 1.35 years of age. Of these, 1,799 children had prospective daycare exposure data. From these, 16 were excluded due to missing breastfeeding duration or ethnicity information, leaving 1,783 children in the analysis cohort. The analysis cohort included 58 children who developed T1D during follow-up of an average of 8.5 years (range 0.9–17.4 years). Three children developed type 1 diabetes before 2 years of age (at ages 0.9, 1.8, and 1.9 years). In these instances, only the information regarding daycare attendance prior to the development of diabetes was used to determine their daycare exposure variable.

### 2.6. Statistical Analysis

The SAS version 9.3 (SAS Institute Inc.) statistical software package was used for all statistical analyses. Hazard ratios (HR) and 95% confidence intervals (CI) were estimated using Cox regression, to account for right-censored data. Follow-up time began at birth. A clustered time to event analysis was performed treating siblings from the same family as clusters, and robust sandwich variance estimates were used for statistical inference [13]. Based on our* a priori* hypothesis, we tested the significance of an interaction between the dichotomous daycare attendance variable and continuous breastfeeding duration variable; interaction models contained the base terms and the interaction term. The significance of the interaction term was determined by improvement in model fit as indicated by the chi-squared statistic from the likelihood ratio test.

## 3. Results

Children who developed T1D in the analysis cohort were more likely to have the HLA-DR3/4, DQB1^*^0302 genotype and a father or sibling with T1D (Table 1). Being non-Hispanic white was marginally associated with an increased T1D risk. Univariately, daycare attendance and breastfeeding duration were not associated with T1D risk (Table 1). After adjusting for HLA, first degree relative with T1D, ethnicity, and breastfeeding duration, attending daycare during the first two years of life was not associated with the risk of developing T1D (HR: 0.89; CI: 0.54–1.47, *P* value = 0.64), while each additional month of breastfeeding duration was associated with a 5% decreased risk of developing T1D (HR: 0.95; CI: 0.90–1.00, *P* value = 0.05).

**Figure 1:**
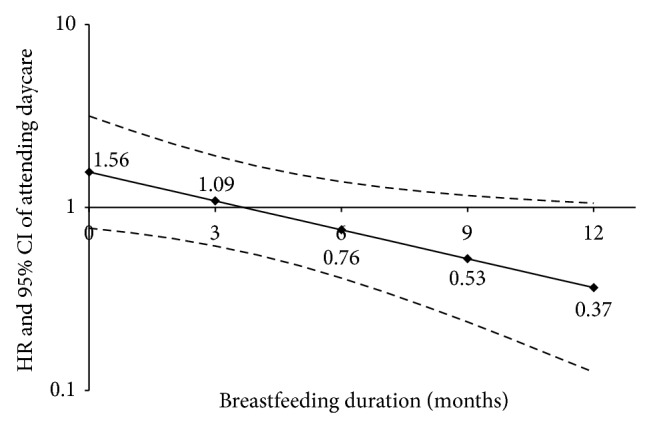
The association between attending daycare in the first 2 years of life and risk of developing T1D in children who were breastfed for 0 months (i.e., not breastfed), 3 months, 6 months, 9 months, and 12 months. The HRs and 95% CIs are calculated from the interaction term, daycare ∗ breastfeeding duration (as a continuous variable), in the model adjusting for HLA, first degree relative with T1D, and ethnicity (interaction *P* value = 0.02).

We* a priori* hypothesized that breastfeeding would modify the effect of attending daycare on the risk of developing T1D. Our analyses showed that breastfeeding duration interacted with daycare attendance, where daycare attendance was associated with increased risk of T1D in nonbreastfed children and a decreasing risk of T1D with increasing breastfeeding duration (interaction *P* value = 0.02) (Table 2). To demonstrate this relationship, we calculated HR estimates and 95% CI for daycare attendance for 0, 3, 6, 9, and 12 months of breastfeeding duration (Figure 1). The highest risk of developing T1D was observed in children who attended daycare and were not breastfed (HR: 1.56; CI: 0.77–3.16), and the lowest risk of T1D was observed in children who attended daycare and were breastfed for 12 months (HR: 0.37; CI: 0.13–1.06).

## 4. Discussion

We found that breastfeeding modified the effect of daycare, where daycare attendance was associated with increased risk of T1D in nonbreastfed children and a decreasing risk of T1D with increasing breastfeeding duration. These findings lend support to both the trigger-booster hypothesis and the hygiene hypothesis. The trigger-booster hypothesis argues that the immunologic trigger in the natural history of T1D is an infection, such as an enterovirus infection. This infection then triggers the autoimmune response that progresses towards overt diabetes [14]. The Eurodiab Substudy 2 showed that reported infections early in a child's life, noted in the hospital record, were found to be associated with an increased risk of T1D (i.e., evidence for the trigger-booster hypothesis); however, preschool/daycare attendance used as a proxy to measure total infections in early childhood was found to be inversely associated with diabetes [15], suggestive of the hygiene hypothesis. Our findings of an increased risk of T1D for attending daycare in the absence of breastfeeding support the trigger-booster hypothesis that daycare may be increasing exposure to diabetogenic infections that are triggering the development of autoimmunity. The decreased risk associated with daycare attendance in breastfed children supports the hygiene hypothesis, suggesting that breastfeeding is providing immunological support to fight off diabetogenic infections while daycare provides an environment that stimulates the immune system with nonspecific infections preventing immune responses against self-antigens. These findings suggest that breastfeeding may be required to glean the benefits of the daycare environment. In sum, breastfeeding may provide the immune support to fight off diabetogenic infections, while allowing the low immune stimulation found in daycare environments to prevent the development of autoimmunity and T1D.

One limitation to using daycare as a proxy for infections is that it does not account for the effects of specific infections, as some infections have been associated with increased risk of T1D development and this detail is lost in using daycare as a proxy for all infections [16]. Furthermore, our questionnaire data lacked the level of detail to calculate duration or intensity of daycare exposure; therefore, this study could not evaluate a dose-response relationship between amount of time in daycare and risk of developing T1D. A strength of the study is that the data were collected prospectively, increasing the accuracy. However, the small number of children with T1D may limit the inference.

The presence of the interaction between daycare attendance and breastfeeding duration suggests a complex interplay between exposures in the etiology of T1D and may explain, in part, the difficulty in identifying environmental risk factors for the disease. Due to the small number of children with T1D in our analysis cohort, our findings should be confirmed in other populations. Future analyses examining environmental exposures in the risk of T1D should hypothesize and test biologically plausible effect modifications such as the one identified here, in order to more clearly elucidate the etiology of the T1D.

## Figures and Tables

**Table 1:** Characteristics of the analysis cohort by T1D status (the Diabetes Autoimmunity Study in the Young).

	Number (%)		Univariate HR (95% CI)	*P* value
	Developed T1D (*n* = 58)	Did not develop T1D (*n* = 1725)		
Age (mean (SD) at T1D diagnosis or at last followup, years)	8.5 (3.8)	8.8 (5.6)	N.A.	N.A.
HLA-DR3/4, DQB1^*^0302	32 (55.2)	412 (23.9)	3.35 (1.99–5.64)	<0.0001
First degree relative with T1D				
Mother	4 (6.9)	174 (10.1)	1.08 (0.37–3.14)	0.88
Father or sibling	32 (55.2)	390 (22.6)	3.50 (1.99–6.17)	<0.0001
Birth order (first/only child)	20 (36.4)	712 (43.3)	0.78 (0.45–1.38)	0.40
Female	27 (46.5)	836 (48.5)	0.93 (0.54–1.60)	0.80
Race/ethnicity (non-Hispanic white)	50 (86.2)	1202 (69.7)	2.10 (0.92–4.81)	0.08
Maternal age, mean (SD), years	30.6 (5.9)	30.0 (5.7)	1.00 (0.95–1.05)	0.97
Maternal education (>12 years)	41 (70.7)	1255 (75.6)	0.62 (0.35–1.12)	0.11
Crowding (≥1 people/room at 6 mo.)	6 (10.3)	227 (13.2)	1.05 (0.45–2.47)	0.91
Ever breastfed	54 (93.1)	1483 (86.0)	1.84 (0.67–5.03)	0.23
Breastfeeding duration, mean (SD), months	5.6 (6.9)	6.4 (7.0)	0.96 (0.91–1.02)	0.16
Daycare attendance in the first 2 years of life	27 (46.5)	803 (46.5)	0.89 (0.53–1.49)	0.65

**Table 2:** Association between daycare attendance and risk of developing T1D (the Diabetes Autoimmunity Study in the Young).

Variable	Adjusted HR	95% CI	*P* value

HLA-DR3/4, DQB1^*^0302	5.06	(2.95–8.69)	<0.0001
First degree relative with T1D			
Mother	1.80	(0.64–5.06)	0.27
Father or sibling	4.79	(2.60–8.84)	<0.0001
Race/ethnicity (non-Hispanic white)	1.95	(0.78–4.85)	0.15
Breastfeeding duration, months	∗	∗	0.38
Daycare attendance in the first 2 years	∗	∗	0.21
Breastfeeding duration ∗ daycare attendance in the first 2 years of of life	∗	∗	0.02

^*^The HRs and CIs of the breastfeeding duration and day care attendance in first 2 years variables were not calculated as these variables were components of the significant interaction term. The interaction between these variables is depicted in Figure 1.
